# Goal-directed vs. habitual instrumental behavior during reward processing in anorexia nervosa: an fMRI study

**DOI:** 10.1038/s41598-019-49884-6

**Published:** 2019-09-19

**Authors:** Julius Steding, Ilka Boehm, Joseph A. King, Daniel Geisler, Franziska Ritschel, Maria Seidel, Arne Doose, Charlotte Jaite, Veit Roessner, Michael N. Smolka, Stefan Ehrlich

**Affiliations:** 1 Division of Psychological and Social Medicine and Developmental Neurosciences, Faculty of Medicine, TU Dresden, Dresden, Germany; 2 Department of Child and Adolescent Psychiatry, Psychosomatics and Psychotherapy, Charité – Universitätsmedizin Berlin, corporate member of Freie Universität Berlin, Humboldt-Universität zu Berlin and Berlin Institute of Health, Berlin, Germany; 3 Department of Child and Adolescent Psychiatry, University Hospital Carl Gustav Carus, TU Dresden, Dresden, Germany; 4 Department of Psychiatry and Neuroimaging Center, TU Dresden, Dresden, Germany; 5 Translational Developmental Neuroscience Section, Department of Child and Adolescent Psychiatry, Eating Disorders Research and Treatment Center, University Hospital Carl Gustav Carus, TU Dresden, Dresden, Germany

**Keywords:** Reward, Psychiatric disorders

## Abstract

Previous studies have proposed that altered reward processing and elevated cognitive control underlie the etiology of anorexia nervosa (AN). A newly debated notion suggests altered habit learning and an overreliance on habits may contribute to the persistence of AN. In weight-recovered AN patients, we previously found neuroimaging-based evidence for unaltered reward processing, but elevated cognitive control. In order to differentiate between state versus trait factors, we here contrast the aforementioned hypotheses in a sample of acutely underweight AN (acAN) patients. 37 acAN patients and 37 closely matched healthy controls (HC) underwent a functional MRI while performing an established instrumental motivation task. We found no group differences with respect to neural responses during the anticipation or receipt of reward. However, the behavioral response data showed a bimodal distribution, indicative for a goal-directed (gAN) and a habit-driven (hAN) patient subgroup. Additional analyses revealed decreased mOFC activation during reward anticipation in hAN, which would be in line with a habit-driven response. These findings provide a new perspective on the debate regarding the notion of increased goal-directed versus habitual behavior in AN. If replicable, the observed dissociation between gAN and hAN might help to tailor therapeutic approaches to individual patient characteristics.

## Introduction

Anorexia nervosa (AN) is an eating disorder characterized by a relentless pursuit of thinness and has one of the highest mortality rates in psychiatry^1^. Although the etiology is still unclear, there is a consensus that neurobiological processes play a role in the development and maintenance of the disorder^2,3^. Recent theoretical models include the notion of an overactive “top-down” cognitive control system that inhibits or compensates for altered “bottom-up” appetitive signaling and alterations in the brain reward system in AN^4–6^. Such models offer an explanation not only for patients’ ability to suppress the drive to eat despite undernourishment, but also the anhedonic response to other primary rewards such as sex and pleasant touch^7,8^.

The “top-down” cognitive control system is associated with fronto-parietal brain regions (in particular dorsolateral prefrontal cortex, dlPFC) and is crucial when controlling responses to rewarding stimuli including food^9^. Jensen *et al*.^10^ found increased activation in the dlPFC to high-calorie food pictures in obese individuals that succeeded in losing weight. Interestingly, another study^11^ suggested a modulating role of the dlPFC over the medial orbitofrontal cortex (mOFC) during the choice between healthy versus tasty food options. The latter brain region, part of the mesocorticolimbic reward system, has generally been associated with the integration and encoding of (sometimes competing) goal values and is thought to play a crucial role in value-based decision making and goal-directed behavior^12–14^. Another important part of the brain reward system is the ventral striatum (VS), a brain region rich in dopaminergic input^15,16^.

DlPFC, mOFC and VS are of relevance during cognitive control and the processing of rewarding stimuli (including food) and have therefore been subject of recent imaging research in AN^6,17^. Regarding the VS, several but not all studies indicate aberrant neural responses in AN during reward processing, but the direction of the effect is somewhat heterogeneous. Some studies found increased activation in the VS during the processing of monetary rewards^18^, taste stimuli^19^ and underweight body pictures^20^ in acute AN (acAN) as well as recovered AN (recAN) patients^21^. On the other hand, two studies found deactivation in the VS when monetary rewards where presented to acAN^22^ and taste stimuli to recAN patients^23^. As for the mOFC, increased activation was found in both acAN and recAN using food pictures^24^ as well as in acAN using taste stimuli^21^. Interestingly, lateral prefrontal cortex hyperactivation to reward stimuli was predominantly found in acAN when exposing them to taste stimuli^21^, food pictures^25^, body pictures^26^ and exercise cues^27^, which may indicate elevated cognitive control over rewarding stimuli during the acute phase of the disorder. Taken together, the existing literature suggests aberrant reward-related neural responses in mesocorticolimbic regions and exaggerated cognitive control reflected by altered lateral prefrontal cortex responses to rewarding stimuli, especially during the acute phase of AN. However, it remains an open question whether these brain alterations are related to the state of undernutrition during the acute phase of the illness or predisposing traits^28^.

When investigating reward processing, it is important to differentiate between reward anticipation and receipt, as the activity profile of dopaminergic neurons differs^29^. One paradigm used to separate these two components is the instrumental motivation task, a variant of the monetary incentive delay task^30^, which uses response vigor as a behavioral index of motivation and effort^31^. In previous work using this task^32^, some participants showed relatively high effort independent of reward level, suggestive of habitual responding. Habitual behavior is defined as behavior that has been performed repeatedly and subsequently, as a result of stimulus-response learning, occurs with little to no conscious effort, even when the outcome is absent or has changed and is therefore no longer rewarding^33^. Recently, AN has been proposed to be characterized by over-reliance on habit learning and habitual behavior^34^. Restricting food intake may begin as a goal-directed weight-loss behavior associated with a rewarding outcome (e.g. positive social reinforcement to weight loss), which over time and with enough repetitions becomes relatively independent of reward^35^. Indirect support for this theory comes from a study which found elevated activation in the dorsal striatum during a food choice task suggesting that maladaptive food choice behavior may not be due to an elevated amount of self-control, but rather habitual^36^. However, in the first behavioral study to probe this account, AN patients did not show any differences compared to healthy participants in two outcome-devaluation tasks designed to measure the conflict of habit learning with goal-directed behavior^37^. To date it is unclear whether the habit account of AN is an alternative or complementary to the aforementioned “over-control” theory^38^.

In a previous study using the instrumental motivation task in recAN^39^, we found no differences in VS or mOFC activity associated with anticipation or receipt of monetary rewards. However, recAN not only showed stronger dlPFC activation during reward anticipation, but also failed to deactivate this region during the feedback phase. RecAN patients were studied to eliminate potential confounding effects of undernutrition. However, this approach carries the risk of only examining a subgroup of patients who eventually recover^28^. To clarify whether altered reward processing and/or the top-down control thereof is state or trait, we investigated hemodynamic activity in mesolimbic and prefrontal regions in acAN patients in comparison to closely matched healthy controls (HC) during anticipation and receipt of monetary rewards as in our previous study. Additionally, we used an approach similar to Kroemer *et al*.^32^, to test whether patients would adopt a goal-directed or habitual behavioral strategy when solving this task and identified the underlying neural mechanisms.

## Methods

### Participants

The sample of the current study consisted of 74 female volunteers: 37 patients with acAN according to DSM-IV (age 12.4 to 23.3) and 37 female healthy controls (HC; age 12.3 to 23.4).

All patients were admitted to eating disorder programs of a university child and adolescent psychiatry and psychosomatic medicine department and were assessed within 96 hours after beginning a behaviorally-oriented nutritional rehabilitation program. HC participants had to be of normal weight, eumenorrhoeic and without any history of psychiatric illness. HCs were recruited through advertisement among pupils and university students.

We applied several additional exclusion criteria for each group (see Supplemental Materials (SM) 1) – most importantly psychotropic medication within four weeks prior to the study, binge eating, or diagnosis of bulimia nervosa, substance abuse, neurologic or medical conditions. Case-control matching was carried out using the SPSS “Fuzzy” algorithm allowing for a maximum age difference of two years between the individuals within one pair.

This study was approved by the ethics committee of the Technische Universität Dresden and carried out in accordance with the latest version of the Declaration of Helsinki, and all participants (and their guardians if underage) gave written informed consent.

### Clinical Measures

For all participants, the presence or absence of current diagnoses of eating disorders were ascertained by evaluation of the expert form of the Structured interview of anorexia nervosa and bulimia nervosa (SIAB-EX)^40^. Interviews were conducted by clinically experienced and trained research assistants under the supervision of the attending child and adolescent psychiatrist.

In addition to the clinical interviews, eating disorder-specific psychopathology was assessed with the German version of the Eating Disorders Inventory (EDI-2)^41^. Furthermore, depressive symptoms were examined using the depression subscale of the German version of the Symptom Checklist (SCL-90-R)^42^.

IQ was assessed with a short version of the German adaption of the Wechsler Adult Intelligence Scale^43^ or a short version of the German adaption of the Wechsler Intelligence Scale for Children^44^ for participants aged 15 years or younger.

BMI standard deviation scores (BMI-SDS) were calculated for each participant, which is controlled for both age and sex^45^.

### Instrumental Motivation Task

**Figure 1:**
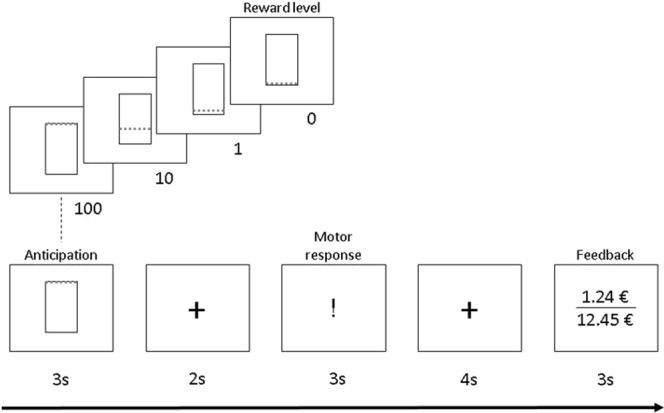
Instrumental motivation task. Instrumental motivation task during event-related functional MRI (fMRI). During the anticipation phase a visual cue was presented for 3 s to inform the participant about the reward level of this trial (reward levels: 0 [no reward], 1, 10, 100). The motor (or instrumental) response phase started after a 2 s fixation period. Monetary reward per trial increased with reward level and higher effort and was determined by multiplying number of button presses × reward level × an individual adjustment factor (calculated based on the individual maximum #bp in the test run; for details see Bühler and colleagues, 2010). Acoustic feedback for button presses was provided through headphones. After another fixation period of 4 s, feedback was provided for 3 s by displaying the amount of money gained in this trial and the cumulative amount. Between trials, participants fixated on crosshairs for 3 s (75%) or 7.44 s in 25% of all trials, which improves design efficiency by jittering. The fMRI main run had a total duration of 15.5 min and comprised 48 trials in total (4 reward levels × 12 pseudorandomized repetitions; SM 2).

During the fMRI session, participants performed the instrumental motivation task^31,32^. In addition to allowing for measurement of event-related brain activity in response to stimuli predicting monetary reward (reward anticipation) and feedback about the magnitude of the reward received, this particular task variant has the advantage of providing behavioral assessment of motivation operationalized as instrumental responding (number of button presses, #bp) to maximize reward^31^. Each trial included an anticipation phase, a motor response phase, and a feedback (receipt) phase (Fig. 1 for details). The scanning session started with an eight-trial test run to determine each individual’s maximum #bp. This information was used to standardize the cumulative monetary gain to ≈€10 in the subsequent main run, irrespective of inter-individual performance differences in motor speed (for more information, see SM 2).

### Instrumental Response Data Analysis

We compared average #bp and reaction times (RT) of initial responses at each reward level between acAN and HC participants using linear mixed-effects models for the analysis of repeated measurement treating participants as random effects. We assumed a compound symmetry covariance structure for changes of instrumental response by reward level (0, 1, 10, 100) and included group (acAN, HC) as a factor as well as an interaction effect (slope) between group and reward level. Since the variable indicating reward level was centered (and HC were used as reference for the factor group) the intercept in HC (and intercept + main effect of group in acAN) represents not instrumental responses at reward level 0 but the “typical” response, i.e. it correlates highly with the response rate at an average reward level.

### Structural and Functional Image Acquisition, Data Processing and Analysis

Images were acquired following an overnight fast between 8 and 9 a.m. using standard sequences with a 3 T whole-body MRI scanner (TRIO; Siemens, Erlangen, Germany) equipped with a standard head coil (SM 3). Functional and structural images were processed with SPM8 (http://www.fil.ion.ucl.ac.uk/spm) within the Nipype framework (http://nipy.sourceforge.net/nipype/)^46^ following standard procedures (SM 4). We evaluated the quality of the fMRI data by manual inspection and using the artifact detection tool (ART)^47^. Volumes that exceed an intensity threshold of three standard deviations or a threshold of 2 mm normalized movement in any direction were classified as outliers (motion-outlier: acAN patients: 2.84 ± 11.62, HC: 3.38 ± 5.96; intensity-outlier: AN patients: 5.43 ± 3.80, HC: 5.14 ± 3.06). The two groups did not differ regarding numbers of motion- and intensity-outliers (motion-outlier: t(72) = 0.25, p = 0.80; intensity-outlier: t(72) = 0.56, p = 0.58).

On the single subject-level a general linear model (GLM) was fit to model the brain activation in response to increasing reward levels. We modeled all four reward levels of the anticipation, motor response phase and feedback phase as single events (12 regressors). Additional regressors included six motion parameters as well as one regressor for each motion or intensity outlier volume (see SM 4).

Exploratory whole-brain group-level analyses to investigate main effects of task and group were performed with SPM8 (www.fil.ion.ucl.ac.uk/spm). To control for false-positives, family-wise error (FWE) correction was performed using the program 3DClustSim (http://afni.nimh.nih.gov/afni; “fixed” version compiled June 2017).

Based on well-established models of the reward system^48,49^ and our research question, we obtained indices of activation for bilateral VS, mOFC, and dlPFC ROIs (see SM 4). Extraction of ROI-specific beta-values for each reward level during anticipation and feedback phase obtained for each participant was performed using the MarsBaR toolbox for SPM^50^. As for instrumental response data (behavioural data, see above), we analysed the extracted indices of neural responses using linear mixed-models.

### Additional Statistical Analyses

In order to test for associations between symptom scores and the magnitude of change in neural responses with increasing reward levels (using Pearson’s r), we modelled the ROI-based beta values in each participant using linear regression analysis (yielding individual intercepts and slopes of neural activity across reward levels) in SPSS v21.0 (SPSS, Chicago, Illinois). Again, the independent variable indicating reward level was centered, i.e. the intercept represents the “typical” response (see above).

## Results

### Sample characteristics

There were no differences between the two pairwise-matched groups regarding age and IQ. As expected, the acAN patients had a lower BMI-SDS as well as higher EDI-2 total and SCL-90-R depression scores (for a detailed description, see Table 1).

**Table 1:** Demographic and clinical characteristics of the sample.

	acAN (*n* = 37)		HC (*n* = 37)		*t*	*p*
	*M*	*SD*	*M*	*SD*		
age	16.01	2.53	16.23	2.64	0.37	0.71
IQ	112.59	11.51	111.44	9.85	0.45	0.66
BMI-SDS	−3.15	1.49	−0.15	0.77	10.9	<0.001
EDI-2 total	196.9	47.55	141.61	29.1	5.85	<0.001
SCL-90-R depression	27.24	11.77	18.54	6.22	3.98	<0.001
duration of illness in years	1.94	2.05	—	—		

*Notes. ac*AN = acute anorexia nervosa, HC = healthy control participants. Group differences were tested using Student’s t-tests. IQ = intelligence quotient, BMI-SDS = body mass index standard deviation score, EDI-2 = eating disorder inventory 2, SCL-90-R = symptom checklist 90 revisited. Of the 37 acAN patients, 35 were of the restrictive and 2 of the binge/purge subtype.

### Instrumental response data

**Figure 2:**
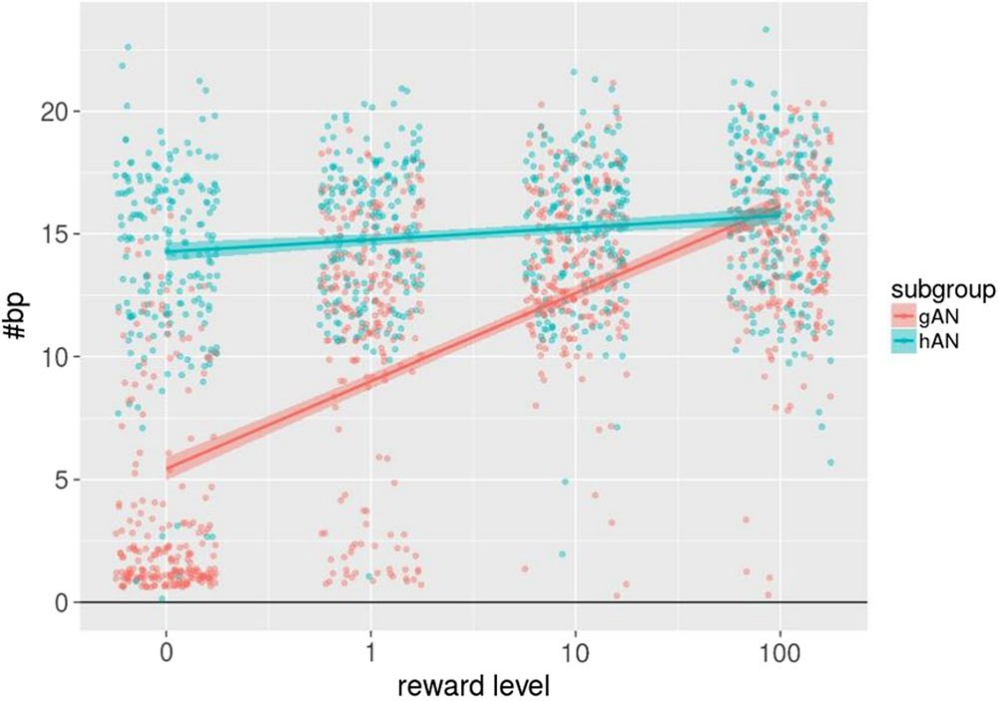
Behavioral data of subgroups. Distribution of raw values of number of button presses (#bp) over all four reward levels of goal-directed (gAN; red dots) and habit-driven AN (hAN; green dots). Additionally, a smooth regression line was added for both subgroups.

RTs decreased (F_(1,222)_ = 176; p < 0.001) and #bp increased (F_(1,222)_ = 183.1; p < 0.001) with each ascending reward level in both groups as expected, but no group differences were evident (both F_(1,74)_ < 1.2, n.s.; for more information, see Table S.1 in SM 5) indicating a similar degree of goal-directedness in both AN and HC. Given the recent debate about the role of goal-directed versus habitual behavior in AN^34,35^, we next aimed to understand if in fact all individuals with AN adopted a goal-directed behavioral strategy when solving this task. Notably, the distribution of #bp per reward level was found to be bimodal (see Fig. 2) with some patients showing a steeper slope in #bp with increasing reward level and some showing a less steep slope.

The less steep slope in a subgroup of acAN participants could be interpreted as a tendency towards habitual behavior (i.e. not modulated by reward level). Given the bimodal distribution, we ran additional analyses by separating the acAN group according to the distribution of #bp into a group with seemingly more goal-directed behavior (gAN) and a second one with seemingly habit-driven behavior (hAN) (see Fig. 2) using a cluster analysis (for further details, see SM 6). This resulted in a gAN group of n = 19 and an hAN group of n = 17. As expected, these groups differed significantly with respect to the #bp slope when the initial mixed model was repeated (see SM 6). However, subgroups were similar in age, IQ, BMI-SDS, duration of illness, SCL-90-R depression score and EDI total score (Table 2).

**Table 2:** Clinical measures within the AN clusters.

	gAN (*n* = 19)		hAN (*n* = 17)		*t*	*p*
	*M*	*SD*	*M*	*SD*		
age	16.45	2.41	15.45	2.69	1.18	0.247
IQ	114.18	9.32	111.00	13.44	0.80	0.429
BMI-SDS	−3.56	1.68	−2.64	1.14	1.90	0.066
duration of illness in years	2.47	2.51	1.36	1.29	1.70	0.101
EDI-2 total score	210.78	46.87	184.24	44.56	1.66	0.108
SCL-90-R depression	31.00	12.58	23.76	9.72	1.91	0.064
min BMI	14.11	1.41	14.81	1.20	1.60	0.119

*Notes*. AN = anorexia nervosa, gAN = goal-directed AN subgroup, hAN = habit-driven AN subgroup. Group differences were tested using Student’s t-tests. BMI-SDS = body mass index standard deviation score, EDI-2 = eating disorder inventory 2, SCL-90-R = symptom checklist 90 revisited, min BMI = minimal lifetime body mass index.

### Neuroimaging data

The results of exploratory whole brain analyses are shown in SM 7 and visualize the main effect of task and confirm expected activation patterns based on previous studies with this task^32,39^. There were no significant group differences (FWE-corrected p = 0.05).

ROI-based analyses using extracted beta values and mixed models confirmed a significant main effect of reward level during reward anticipation in the VS (F_(1,222)_ = 6.94, p = 0.01) indicating a stepwise activation increase in response to increasing reward. Likewise, we found an expected significant main effect of reward level in the left dlPFC during reward feedback (F_(1,222)_ = 7.96, p = 0.001). However, no group differences were evident in any ROI either during reward anticipation or feedback (all F_(1,74)_ < 3.15, n.s.). A detailed description of the results of the linear mixed models can be found in Table S.3 in SM 8. Furthermore, and in contrast to our previous findings in recAN, there were no brain-behavior correlations between #bp and mean activation in the dlPFC.

### Additional analyses

**Figure 3:**
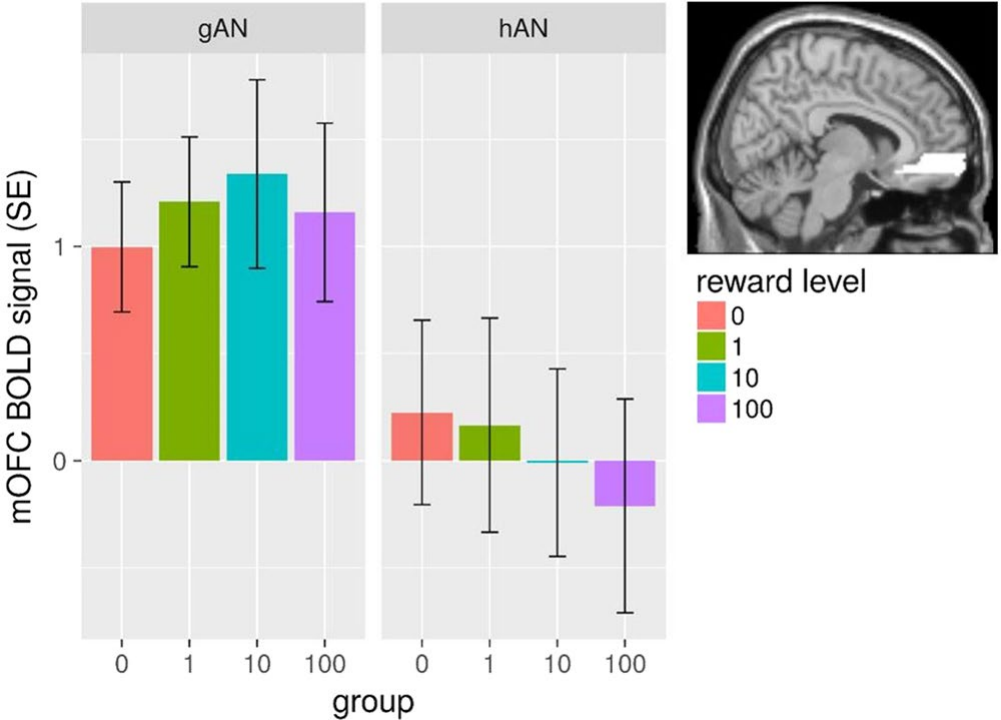
Mean mOFC BOLD signal for subgroups. Mean mOFC BOLD signal for each reward level during anticipation for both goal-directed (gAN) and habit-driven AN (hAN), showing a significant group difference (across reward levels) in a linear mixed model (see results section for details). Error bars depict the standard error of the mean (SE).

Finally, based on the results of the cluster analysis which distinguished between acAN who performed the instrumental motivation task in a more habitual manner (hAN) and those who responded in a more goal-directed fashion (gAN), we repeated the linear mixed models for reward anticipation in all ROIs (see Table S.5 in the SM 9) to explore potential differences in brain activation between these subgroups. Interestingly, although no significant effects were evident in dlPFC or VS, a significant difference was uncovered in the mOFC with gAN showing a significantly higher mean BOLD activity than hAN (F_(1,36)_ = 8.19, p = 0.007; see Fig. 3). To address the question of disorder-specificity, we ran the same analysis in HC which could also be separated in a goal-directed (n = 20) and habit-driven (n = 17) subgroups. However, there were no group differences in any of the ROIs (see SM 10).

## Discussion

In this study we investigated reward processing with respect to goal-directed and habitual behavior in AN. Similar to weight-recovered AN patients^39^, we found no evidence for generally abnormal reward processing in acAN. In contrast, however, alterations found in brain regions related to cognitive control in recAN^39^ were not present in the current acAN sample. However, as part of our analysis aiming to differentiate goal-directed versus habitual behavioral tendencies in AN, we observed a bimodal behavioral response pattern (#bp) within acAN, possibly reflecting two subgroups: namely, one with seemingly habit-driven behavior due to generally high #bp independent of reward level (hAN) and one with goal-directed behavior characterized by increasing #bp with higher reward levels (gAN). Most importantly, when repeating the fMRI analyses with these two subgroups, we found mean mOFC activation during reward anticipation to be higher in gAN compared to hAN. One possible interpretation for this dissociation between AN subgroups in terms of mOFC activation may be enhanced value representation in gAN, which may explain the more adaptive and therefore goal-directed behavior in terms of a favorable cost-benefit ratio^51–53^. This view is supported by a study in healthy individuals showing increased mOFC activation during predominantly goal-directed actions compared to habitual behavior^54^. HAN, in contrast, showed no notable mOFC activation during reward anticipation and it could be speculated that this reflects a rigid or seemingly habit-driven behavioral response that does not rely strongly on value representations. We thus suggest the existence of a subgroup of acAN patients that are characterized by habitual behavior which might have implications for the course and in the maintenance of AN^35,38^. Of note, Gillan and colleagues^55^ showed that eating disorder-related symptomatology in healthy individuals was associated with a habitual approach using a two-step reinforcement learning task designed to measure the relative influence of goal-directed versus habitual learning.

Although the notion of altered habit learning and associated maladaptive behavior in AN is well-established in theory^34,35^, direct empirical evidence is sparse^38^. One approach to study habits in AN is via self-report. In a study by Coniglio *et al*.^56^ using the Self-Report Habit Index (SRHI), anorectic behavior (food restriction) was better explained by habit strength than by cognitive restraint. In another study by Steinglass *et al*.^57^, AN patients showed greater habit strength for self-selected habits related to eating in comparison to HC. The only known experimental study so far found no differences in AN compared to HC regarding the conflict of habitual over goal-directed behavior in an outcome devaluation paradigm^37^, but existing tasks have several limitations^58^. One drawback of the task employed by Godier *et al*.^37^ is that habitual behavior can only be measured when it is in conflict with goal-directed behavior. Last but not least Giannunzio *et al*.^59^ defined two AN subgroups in a study applying the “expectancy-valence” model^60^ to data from the Iowa Gambling Task (IGT). They found a “conservative” subgroup with high consistency and low updating as well as an “impulsive” subgroup with low consistency and high updating. Although the results and model parameters cannot be easily translated to our reward paradigm, the habit-driven subgroup in the current study may potentially be characterized by high consistency and low updating.

Alternative explanations that could account for the observed behavioral pattern in hAN include increased inflexibility or perseveration in AN. Patients suffering from AN have been reported to show preservative and rigid thinking styles^61,62^ as well as reduced cognitive flexibility^63,64^. Notably, cognitive inflexibility has been found in adult AN patients and may be a consequence of longer illness duration^65^, while the results in adolescents have been mixed or less strong^64^. Although our sample was predominantly adolescent, the hAN subgroup could consist of individuals with stronger cognitive inflexibility that respond to each reward level irrespective of reward rather than adjusting each response to the amount of reward. Speculatively, this subgroup might be more prone to a chronic course. Other potential explanations include a social desirability bias^66^ or increased willingness to exert effort independent of the reward magnitude in the hAN subgroup^67^. Another reason for different behavioral response patterns between the subgroups could be genetics. For example, COMT polymorphisms have been linked to dopamine release in prefrontal regions and thus to executive functioning. In a study by Favaro *et al*.^68^, AN patients with the Met-Met COMT genotype showed greater dlPFC and mOFC coactivation than Val genotype carriers. Such mechanisms could potentially be part of the missing link to heterogeneous results regarding executive functioning and reward processing in AN.

In our work with the current task, we found no general impairment of reward processing with respect to dopaminergic mesocorticolimbic regions either in acAN or recAN^39^. This is in line with several (but not all, see above) other targeted studies of reward processing in AN that did not find neural alterations in limbic brain regions in anticipation of or response to reward^69–74^. Similarly, in the current acAN sample we did not find differences in lateral prefrontal activity while other studies have reported such findings^21,25–27^ which have been interpreted as indicative of elevated cognitive control. However, in self-report studies patients suffering from the restrictive type of AN often report less sensitivity to reward^75^ as well as a tendency towards increased self-control^76^. Taken together, the neural underpinning of reward processing in AN is still unclear. The current set of results suggests altered valuation processing may not be a generic feature of AN, but may be strongly dependent on individual factors such as goal-directed vs. habitual response tendencies or illness state. Other variables that may contribute to the heterogeneous results are design factors such as sample size, mode of presentation (e.g. event-related or block design) or type of rewards e.g. food vs. non-food^77^.

Although our sample was moderately-sized and larger than most previous fMRI studies of reward processing in AN, the subgroup analyses were limited in statistical power and the results therefore need to be considered with caution. Nonetheless, while a relative lack of power might explain why we did not observe any clear clinical differences between the two subgroups, we believe the absence of such differences strengthens our conclusions because the results cannot be explained by extraneous factors. Conclusions from comparisons between the current findings in acAN and those from our previous study in recAN^39^ also need to be drawn carefully because the samples were independent and differed in both age and duration of illness. Future studies should therefore include longitudinal observations. Additionally, given that our AN sample consisted mostly of patients of the restrictive subtype, the results cannot be generalized to patients of the binge/purge subtype. Yet another limitation is that although all investigated ROIs showed expected activation patterns indicative of reward sensitivity, a clearly monotonic increase of the BOLD signal as a function of reward level was only observed in the VS and our analyses did not take potential curvilinear response patterns into account. This is especially important with respect to our subgroup finding in the mOFC, a region that has often been shown to have a linear increase in BOLD signal with increasing reward value^78^. Thus, these results have to be interpreted with caution. Last but not least, although the employed instrumental motivation task has been used to make inferences about habitual behavior^32^, it was not originally developed for this specific purpose.

In this study, no general deficit in reward processing in acAN was found, but both behavioral and neural evidence suggests altered valuation processes in a subgroup of patients who showed habitual responding. The findings of this study could provide a new perspective on the ongoing discussions regarding the notion of increased goal-directedness versus increased reliance on habitual behavior in AN^34,38,79^. If the found dissociation between gAN and hAN can be replicated, this information might have implications regarding clinical outcomes and help to better tailor therapeutic approaches to harness adaptive behaviors and ameliorate maladaptive ones.

## Supplementary information

Supplements
