# Supplementary material for: Goal-directed vs. habitual instrumental behavior during reward processing in anorexia nervosa: an fMRI study
Source: Sci Rep. 2019 Sep 19;9:13529. doi: 10.1038/s41598-019-49884-6 (PMC6753148; doi:10.1038/s41598-019-49884-6)
Supplement: Supplementary file 1 — Supplements [file 41598_2019_49884_MOESM1_ESM.docx]

**Goal-directed vs. habitual instrumental behavior during reward processing in anorexia nervosa: an fMRI study**

***Supplementary Material***

Julius Steding, Ilka Boehm, Joseph A. King, Daniel Geisler, Franziska Ritschel, Maria Seidel, Arne Doose, Charlotte Jaite, Veit Roessner, Michael N. Smolka, Stefan Ehrlich

1. Participants

Anorexia nervosa (AN) patients were recruited from specialized eating disorder programs of a university child and adolescent psychiatry and psychosomatic medicine department and diagnosed according to DSM-IV criteria using semi-structured clinical interviews. All AN patients were assessed within 96 hours after the beginning of a behaviorally-oriented nutritional rehabilitation program. Inclusion criteria (e.g. the absence of significant restrictive eating patterns or binge–purge behavior), exclusion criteria (e.g. the use of psychotropic medications) and other possibly confounding variables were obtained using the SIAB-EX and our own semistructured interview. Comorbid diagnoses at the time of treatment were taken according to standard practice from medical records and confirmed by an expert clinician with more than 10 years’ experience after careful chart review (including consideration of medical and psychiatric history, physical examination, routine blood tests, urine analysis and a range of psychiatric screening instruments (e.g., SIAB-EX, EDI-2, SCL-90-R). In the AN group 2 participants had associated psychiatric comorbidity at the time of treatment (1 with selective mutism and 1 with dysthymia, obsessive-compulsive disorder and social phobia).

Healthy controls (HC) were recruited through advertisement among middle school, high school and university students. At the time of our study none of the HC participants had an active psychiatric condition ascertained using a semistructured in-house research interview and the expert version of the SIAB-EX. All interviews were carried out by trained graduate or PhD students (psychology or medicine). HC were further excluded if they had any history of psychiatric illness, a lifetime body mass index (BMI) below the tenth age percentile (if < 18 yr)/BMI below 18.5kg/m² (if > 18 yr), or were currently obese (BMI not over 97th age percentile if < 18 yr; BMI not over 26kg/m² if > 18 yr).

Participants of all study groups were excluded if they had a lifetime history of any of the following clinical diagnoses: organic brain syndrome, schizophrenia, substance dependence, psychosis not otherwise specified, bipolar disorder, bulimia nervosa or binge-eating disorder (or “regular” binge eating, defined as bingeing at least once weekly for 3 or more consecutive months). Further exclusion criteria for all participants were IQ lower than 85; psychotropic medication within 6 weeks prior to the study; current substance abuse; current inflammatory, neurologic or metabolic illness; chronic medical or neurological illness that could affect appetite, eating behaviour, or body weight (e.g., diabetes); clinically relevant anemia; pregnancy; and breastfeeding. If there were any indications of persisting or new psychiatric symptoms each case was discussed with a fully board-certified expert clinician, and assessments were extended if necessary.

Study data were collected and managed using the secure, web-based electronic data capture tool REDCap (Research Electronic Data Capture^1^).

1. Instrumental motivation task

This task variant also provides behavioral assessment of motivation operationalized as instrumental responding to maximize reward^2,3^. Instrumental responding is measured via the number of button presses and reaction times (RT) in the motor (or instrumental) response phase of the task. RT was defined as the time elapsing between the beginning of the appearance of the visual stimulus (exclamation mark) and the beginning of a participant’s reaction to it (first button press) in milliseconds.

Before the scanning session, participants completed a practice version of the task (12 trials) to learn how to perform it.

1. Structural and functional image acquisition

Structural and functional MRI data were acquired with a 3T scanner (Magnetom Trio, Siemens).

The T1-weighted structural brain scans were acquired with rapid acquisition gradient echo (MP-RAGE) sequence with the following parameters: number of slices = 176; repetition time (TR) 1900 ms; echo time (TE) 2.26 ms; flip angle 9°; slice thickness 1 mm; voxel size 1 × 1 × 1 mm³; field of view (FOV) 256 × 224 mm²; bandwidth 200 Hz/pixel. The functional images were acquired using gradient-echo T2*-weighted echo planar imaging (EPI) with the following parameters: tilted 17° from anterior–posterior commissure line toward coronal (to reduce signal dropout in orbitofrontal regions); number of volumes = 396; number of slices = 42; TR 2410 ms; TE 25 ms; flip angle 80°; 3 mm inplane resolution; slice thickness 2 mm (1 mm gap resulting in a voxel size of 3 × 3 × 2 mm³); FOV 192 × 192 mm²; bandwidth 2112 Hz/pixel.

1. Functional image data processing and analysis

The slice time corrected functional EPI data were 4D-realigned and registered to their mean. The 6 realignment parameters, characterizing the rigid-body movement (x,y,z, pitch, roll, yaw), were saved and later used as nuisance covariates to account for the variance due to motion. Subsequently these images were coregistered to the participant’s structural brain image. A DARTEL template was created using structural images from all participants^4^. The EPI volumes were then normalized to Montreal Neurological Institute space using the DARTEL template and corresponding flow field. The resulting data were smoothed with an isotropic 8 mm full-width at half-maximum Gaussian kernel. During the image data processing at the single participant-level using a general linear model (GLM), all regressors were convolved with a synthetic hemodynamic response function as implemented in SPM8. To extract data from single participant-level contrast maps we used a priori regions of interest (ROI). The mask of the ventral striatum was specified by binarizing a probabilistic map^5^ with a threshold value of 0.4. The mOFC mask was created by merging the left and right frontal medial orbital cortex label from the Automated Anatomical Labelling (AAL) atlas provided in the Wake Forest University (WFU) PickAtlas for SPM^6,7^. For the left- and right-hemisphere dlPFC masks we merged the regions of superior and middle frontal gyrus in the WFU PickAtlas for the left and the right hemisphere, respectively, but excluded posterior parts with y < 24 in MNI space^8^ (as in our previous work, see ^9,10^).


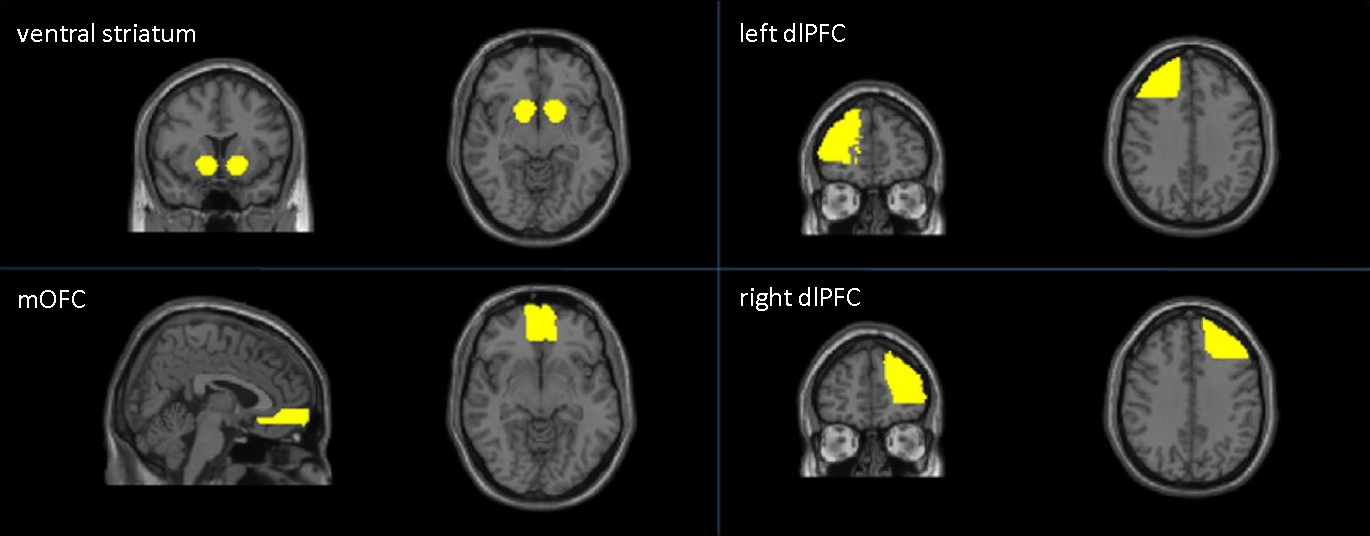


**Figure S.1.** Binarized masks of the investigated regions of interest.

Analogue to the analysis strategy applied to the behavioural instrumental response data, we analyzed the extracted indices of neural responses using linear mixed models in SPSS version 21.0. We assumed a compound symmetry covariance structure for the reward level–dependent change of the hemodynamic response (0, 1, 10, 100 — all centered, see Methods) and included a main effect of group (AN and HC as a factor with control as a reference) as well as an interaction effect (slope) between group and reward level.

1. Results of the linear mixed-effects models of the behavioral data for acAN and HC

| **Table S.1.** Results of the linear mixed-effects models of the behavioral data | | | |
| --- | --- | --- | --- |
| dependent variable | parameter | F statistics | p value |
| #bp | reward level | 183.08 | <.001 |
|  | group | 0.003 | 0.95 |
|  | reward level x group | 1.37 | 0.24 |
|  |  |  |  |
| RT | reward level | 176.04 | <.001 |
|  | group | 1.11 | 0.30 |
|  | reward level x group | 0.02 | 0.90 |
| *Notes.* #bp = number of button presses, RT = reaction times | | | |

1. Description of the cluster analysis

In order to identify the underlying clusters based on #bp data of the patients, we performed a hierarchical cluster analysis with the squared Euclidian distance of the #bp per reward level. Because of the bimodal distribution, we a priori set the number of clusters to 2. The cluster solution resulted in 19 patients characterized by a predominantly goal-directed button response behavior, while 17 patients showed a habit-driven response behavior. One patient could not be classified correctly and had to be excluded.

We performed the aforementioned linear mixed models for both #bp and RTs with the two AN subgroups as independent groups (see Table S.2). As expected, both subgroups differed significantly with respect to mean #bp as well the #bp slope (see Table S.2).

| **Table S.2.** Results of the linear mixed-effects models of the behavioral data for the two AN subgroups. | | | |
| --- | --- | --- | --- |
| dependent variable | parameter | F statistics | p value |
| #bp | reward level | 119.53 | <.001 |
|  | subgroup | 39.68 | <.001 |
|  | reward level x subgroup | 68.18 | <.001 |
|  |  |  |  |
| RT | reward level | 104.33 | <.001 |
|  | subgroup | 3.40 | 0.074 |
|  | reward level x subgroup | 18.10 | <.001 |
| *Notes.* AN = anorexia nervosa, #bp = number of button presses, RT = reaction times. | | | |

1. Whole brain analyses


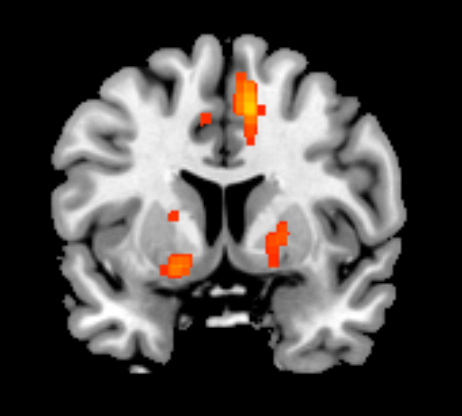


**Figure S.2.** Main effect of task (linear increase of reward level) during anticipation phase for all participants (y=5; p = .001 uncorrected for illustrative purposes).

1. Results of the linear mixed models ROI analyses

| **Table S.3.** Results of the linear mixed-effects models. | | | | |  |  |
| --- | --- | --- | --- | --- | --- | --- |
| ROI | dependent variable | parameter | F statistics | p value | | |
| right dlPFC | anticipation | reward level | 0.58 | 0.45 | | |
|  |  | group | 0.28 | 0.60 | | |
|  |  | reward level x group | 0.24 | 0.62 | | |
|  |  |  |  |  | | |
|  | feedback | reward level | 0.68 | 0.41 | | |
|  |  | group | 1.58 | 0.21 | | |
|  |  | reward level x group | 2.00 | 0.16 | | |
|  |  |  |  |  | | |
| left dlPFC | anticipation | reward level | 1.05 | 0.31 | | |
|  |  | group | 0.01 | 0.93 | | |
|  |  | reward level x group | 0.08 | 0.77 | | |
|  |  |  |  |  | | |
|  | feedback | reward level | 7.96 | 0.005 | | |
|  |  | group | 0.42 | 0.52 | | |
|  |  | reward level x group | 0.71 | 0.4 | | |
|  |  |  |  |  | | |
| VS | anticipation | reward level | 6.94 | 0.01 | | |
|  |  | group | 0.45 | 0.51 | | |
|  |  | reward level x group | 0.05 | 0.82 | | |
|  |  |  |  |  | | |
|  | feedback | reward level | 3.33 | 0.07 | | |
|  |  | group | 0.27 | 0.6 | | |
|  |  | reward level x group | 1.37 | 0.24 | | |
|  |  |  |  |  | | |
| mOFC | anticipation | reward level | 0.001 | 0.98 | | |
|  |  | group | 0.09 | 0.77 | | |
|  |  | reward level x group | 0.11 | 0.74 | | |
|  |  |  |  |  | | |
|  | feedback | reward level | 0.17 | 0.68 | | |
|  |  | group | 3.15 | 0.08 | | |
|  |  | reward level x group | 0.04 | 0.84 | | |
| *Notes.* ROI = region of interest, dlPFC = dorsolateral prefrontal cortex, VS = ventral striatum, mOFC = medial orbitofrontal cortex. | | | | | |  |

To control for effects of the SCL-90-R depression score, we repeated the linear mixed-effects models but found no differences to the original models.

| **Table S.4.** Results of the linear mixed-effects models. | | | | |  |  |
| --- | --- | --- | --- | --- | --- | --- |
| ROI | dependent variable | parameter | F statistics | p value | | |
| right dlPFC | anticipation | reward level | 0.58 | 0.45 | | |
|  |  | group | 0.12 | 0.73 | | |
|  |  | reward level x group | 0.24 | 0.62 | | |
|  |  | SCL-90-R depression | 3.81 | 0.06 | | |
|  |  |  |  |  | | |
|  | feedback | reward level | 0.68 | 0.41 | | |
|  |  | group | 1.90 | 0.17 | | |
|  |  | reward level x group | 2.00 | 0.16 | | |
|  |  | SCL-90-R depression | 0.32 | 0.58 | | |
|  |  |  |  |  | | |
| left dlPFC | anticipation | reward level | 1.05 | 0.31 | | |
|  |  | group | 0.47 | 0.50 | | |
|  |  | reward level x group | 0.08 | 0.77 | | |
|  |  | SCL-90-R depression | 3.26 | 0.08 | | |
|  |  |  |  |  | | |
|  | feedback | reward level | 7.96 | 0.005 | | |
|  |  | group | 1.08 | 0.30 | | |
|  |  | reward level x group | 0.71 | 0.40 | | |
|  |  | SCL-90-R depression | 1.11 | 0.30 | | |
|  |  |  |  |  | | |
| VS | anticipation | reward level | 6.94 | 0.009 | | |
|  |  | group | 0.91 | 0.35 | | |
|  |  | reward level x group | 0.05 | 0.82 | | |
|  |  | SCL-90-R depression | 0.66 | 0.42 | | |
|  |  |  |  |  | | |
|  | feedback | reward level | 3.33 | 0.07 | | |
|  |  | group | 0.33 | 0.57 | | |
|  |  | reward level x group | 1.37 | 0.24 | | |
|  |  | SCL-90-R depression | 0.06 | 0.82 | | |
|  |  |  |  |  | | |
| mOFC | anticipation | reward level | <0.01 | 0.98 | | |
|  |  | group | <0.01 | 0.99 | | |
|  |  | reward level x group | 0.11 | 0.74 | | |
|  |  | SCL-90-R depression | 0.46 | 0.50 | | |
|  |  |  |  |  | | |
|  | feedback | reward level | 0.17 | 0.68 | | |
|  |  | group | 4.09 | 0.047 | | |
|  |  | reward level x group | 0.04 | 0.84 | | |
|  |  | SCL-90-R depression | 0.92 | 0.34 | | |
| *Notes.* ROI = region of interest, SCL-90-R = symptom checklist 90 revisited, dlPFC = dorsolateral prefrontal cortex, VS = ventral striatum, mOFC = medial orbitofrontal cortex. | | | | | |  |

1. Results of the linear mixed models of the gAN and hAN subgroups

| **Table S.5.** Results of the linear mixed-effects models for both the gAN and hAN subgroups during the anticipation phase. | | | |  |
| --- | --- | --- | --- | --- |
| ROI | parameter | F statistics | p value | |
| right dlPFC | reward level | 0.446 | 0.506 | |
|  | subgroup | 0.415 | 0.523 | |
|  | reward level x subgroup | 0.832 | 0.364 | |
|  |  |  |  | |
| left dlPFC | reward level | 0.394 | 0.532 | |
|  | subgroup | 0.109 | 0.743 | |
|  | reward level x subgroup | <0.001 | 0.989 | |
|  |  |  |  | |
| VS | reward level | 3.792 | 0.054 | |
|  | subgroup | 3.502 | 0.069 | |
|  | reward level x subgroup | 1.171 | 0.282 | |
|  |  |  |  | |
| mOFC | reward level | 0.163 | 0.687 | |
|  | subgroup | 8.185 | **0.007** | |
|  | reward level x subgroup | 0.943 | 0.334 | |
| *Notes.* gAN = goal-directed anorexia nervosa patients, hAN = habit-driven anorexia nervosa patients, ROI = region of interest, dlPFC = dorsolateral prefrontal cortex, VS = ventral striate, mOFC = medial orbitofrontal cortex. Reported p values are uncorrected, but only significant p values surviving Bonferroni correction for all four ROIs were marked bold. | | | |  |

1. Results of additional linear mixed models with mOFC response during reward anticipation as outcome

| **Table S.6.** Results of the linear mixed-effects models for the mOFC. | | |
| --- | --- | --- |
| Group effect | F statistics | p value |
| hHC / gHC | 0.837 | 0.366 |
| hAN / HC | 2.564 | 0.115 |
| gAN / HC | 1.808 | 0.184 |
| *Notes.* mOFC = medial orbitofrontal cortex, hHC = habit-driven healthy control participants, gHC = goal-directed healthy control participants, hAN = habit-driven patiens with anorexia nervosa, gAN = goal-directed patients with anorexia nervosa. | | |


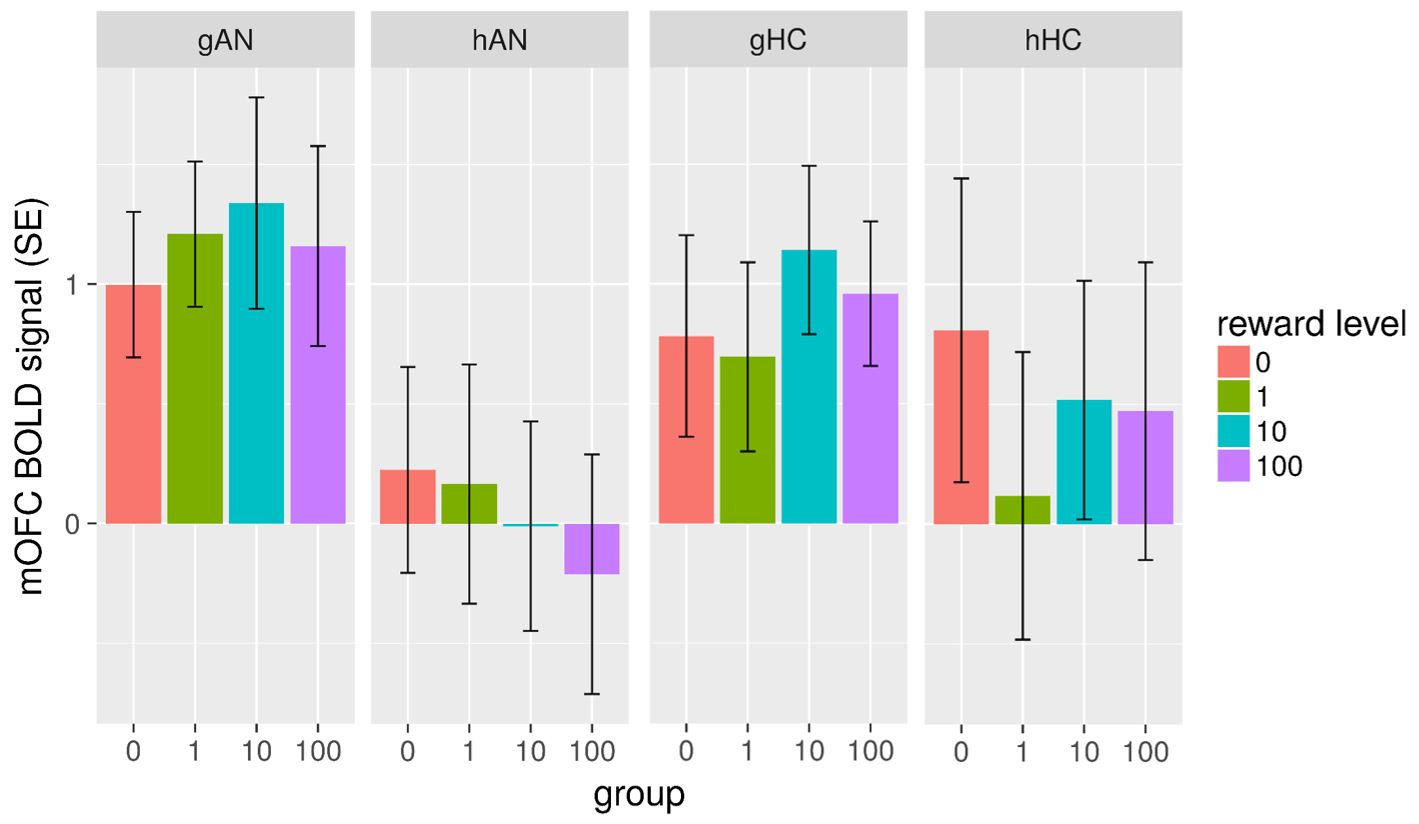


**Figure S.3.** Mean mOFC BOLD signal for each reward level during reward anticipation for both diagnostic groups and the subgroups. Error bars depict the standard error of the mean (SE).

**Literature**

1. Harris, P. A. *et al.* Research electronic data capture (REDCap)—A metadata-driven methodology and workflow process for providing translational research informatics support. *J. Biomed. Inform.* **42**, 377–381 (2009).

2. Bühler, M. *et al.* Nicotine Dependence Is Characterized by Disordered Reward Processing in a Network Driving Motivation. *Biol. Psychiatry* **67**, 745–752 (2010).

3. Kroemer, N. B. *et al.* Balancing reward and work: Anticipatory brain activation in NAcc and VTA predict effort differentially. *NeuroImage* **102**, 510–519 (2014).

4. Ashburner, J. A fast diffeomorphic image registration algorithm. *NeuroImage* **38**, 95–113 (2007).

5. Nielsen, F. A. & Hansen, L. K. Automatic anatomical labeling of Talairach coordinates and generation of volumes of interest via tbe BrainMap database. in (2002).

6. Lancaster, J. L., Summerlin, J. L., Rainey, L., Freitas, C. S. & Fox, P. T. The Talairach Daemon, a database server for Talairach atlas labels. *NeuroImage* **5**, S633 (1997).

7. Lancaster, J. L. *et al.* Automated Talairach atlas labels for functional brain mapping. *Hum. Brain Mapp.* **10**, 120–131 (2000).

8. Ehrlich, S. *et al.* The COMT Val108/158Met polymorphism and medial temporal lobe volumetry in patients with schizophrenia and healthy adults. *Neuroimage* **53**, 992–1000 (2010).

9. Ehrlich, S. *et al.* Striatal function in relation to negative symptoms in schizophrenia. *Psychol. Med.* **42**, 267–282 (2012).

10. Ehrlich, S. *et al.* Elevated cognitive control over reward processing in recovered female patients with anorexia nervosa. *J. Psychiatry Neurosci.* **40**, 307–315 (2015).
